# Death anxiety, exposure to death, mortuary preferences, and religiosity in five countries

**DOI:** 10.1038/s41597-019-0163-x

**Published:** 2019-08-21

**Authors:** Jonathan Jong, Jamin Halberstadt, Matthias Bluemke, Christopher Kavanagh, Christopher Jackson

**Affiliations:** 1 Centre for the Study of Social Cohesion, University of Oxford, Oxford, UK; 2 Department of Psychology, University of Otago, Dunedin, New Zealand; 3 GESIS—Leibniz Institute for the Social Sciences, Mannheim, Germany

**Keywords:** Anxiety, Human behaviour

## Abstract

We present three datasets from a project about the relationship between death anxiety and religiosity. These include data from 1,838 individuals in the United States (n = 813), Brazil (n = 800), Russia (n = 800), the Philippines (n = 200), South Korea (n = 200), and Japan (n = 219). Measures were largely consistent across samples: they include measures of death anxiety, experience of and exposure to death, religious belief, religious behaviour, religious experience, and demographic information. Responses have also been back-translated into English where necessary, though original untranslated data are also included.

## Abstract (summary)

**Table:** 

Design Type(s)	crowd-sourcing design • data integration objective • subject-based data analysis objective
Measurement Type(s)	anxiety
Technology Type(s)	survey method
Factor Type(s)	sex • geographic location • Marital Status • Ethnic Group • age
Sample Characteristic(s)	Homo sapiens • United States of America • Brazil • Russia • The Philippines • South Korea • Japan

Machine-accessible metadata file describing the reported data (ISA-Tab format)

## Background & Summary

There has been long-standing interest in the correlates of death anxiety, including in sex differences, age and cohort effects, effects of exposure to death, and relationships with religiosity. There have been many previous studies on convenience samples (e.g., undergraduate students) and special populations, such as terminally ill patients^1,2^, healthcare and funeral industry professionals^3,4^, and older adults^5,6^; however, studies on the general population are still relatively rare. Furthermore, although—unlike many other aspects of psychological science—there have been several efforts to collect data outside of the West^7,8^, it is often difficult to compare findings across studies due to differences in the way constructs are defined and measured: only a few large-scale multi-national studies stand out in this regard^9^. The intention of this project was to provide more data about death anxiety, religiosity, and other sociodemographic variables that are amenable to cross-cultural hypothesis testing.

The project for which this data was originally collected concerned the relationship between supernatural belief and death anxiety. Two of the scales included in these datasets were developed for the project: the Supernatural Belief Scale and the Existential Death Anxiety Scale, whose initial psychometric evaluations have been published elsewhere^10–12^, and will briefly be described below. This research was designed in accordance with the regulations of the Central University Research Ethics Committee (CUREC) of Oxford University, and the University of Otago Human Ethics Committee, and has been reviewed and approved by these bodies (Ref. SAME/CUREC1A/14-57 and DP:31/14, respectively). Informed consent was obtained for all participants.

## Methods

### Dataset 1: amazon mechanical turk (USA)

Dataset 1 consists of responses from 813 individuals. The study was uploaded to the Qualtrics platform and advertised on Amazon Mechanical Turk between February and May 2015; participants were paid US$1 for completing the survey. We had aimed for N = 800. To achieve this, a total of 892 individuals attempted to participate: 27 did not provide consent for use of data; 13 further failed the attention check; 39 further did not complete the survey. The attention check was placed as the last question of the first page, which asked for sociodemographic information. It was disguised as a Positive and Negative Affect Schedule^13^ (PANAS): instead of selecting options to indicate how they felt, participants were asked to only check the “none of the above” option.

The questionnaires—which can be downloaded in full from the Open Science Framework^14^—contain five sections, always presented in the following order:

1. **Demographics**. We collected data on gender, age, geography, ethnicity, marital status, and employment status/occupation. Previous research on demographic correlates—especially sex differences and age/cohort effects—have produced inconsistent results^5,15^; a few studies have also attempted to explore more sophisticated analyses, including nonparametric (e.g., loess curve fitting^16^) analyses and interaction analyses of different combinations of demographic variables (e.g., sex and age^11^; sex and marital status^17^), which are also possible in this dataset.
2. **Fear of death**. We included four main measures of death-related thoughts and feelings, always in the following order: (1) a free-list measure of participants’ fears and worries, which may be coded; (2) a measure of death thought accessibility^18,19^; (3) Conte, Weiner, and Plutchik’s Death Anxiety Questionnaire (DAQ)^20^; and (4) Jong and Halberstadt’s Existential Death Anxiety Scale (EDAS)^10^. The free-list measure asked participants about what they were “most afraid or most worried about”, instructing them to list their “five biggest fears and anxieties”. The death thought accessibility measure comprised a 25-item word fragment completion task, of which 6 could be completed in either a death-related or death-unrelated manner (e.g., COFF_ _ can be completed as COFFIN or COFFEE). The DAQ is a 15-item multidimensional scale that measures fears related to suffering, loneliness, the unknown, and personal extinction. The EDAS is a 12-item measure of death anxiety that targets the fear of nonexistence: however, this dataset includes an earlier 17-item version, from which 5 were eventually dropped on the basis of psychometric evaluations. Items of the DAQ and EDAS were presented in randomised order. This section concludes with questions about participants’ subjective age, how old they feel, and their predictions of how much longer they will live.
3. **Exposure to death**. This section asks questions about participants’ personal experiences of and exposures to death and dying: including witnessing the deaths of family members, participating in hunting, involvement in armed conflict, and nearly dying themselves. Participants were also asked to provide subjective ratings of how much contact they have with death, the dead, and dying, as well as of their familiarity with the dying process. Finally, participants were asked about their homes’ proximity to cemeteries. Previous research has suggested that exposure to and familiarity with death might shape emotional responses to death^21,22^; this data can speak to this hypothesis and other related hypotheses.
4. **Mortuary preferences**. This section includes questions about participants’ attitudes toward practical preparations for death (e.g., “How concerned are you with what happens to your body after you die?”), followed by questions about preferences for specific mortuary arrangements (e.g., burial, cremation, organ donation, cryonics).
5. **Religiosity**. Religiosity measures included subjective self-ratings of religiosity and spirituality; a question about religious affiliation; rating of importance of religious beliefs; Jong and Halberstadt’s 6-item Supernatural Belief Scale; questions about frequency of religious behaviours, and questions about spiritual experiences. The variety of measures permits researchers to distinguish between different aspects of religiosity to test more specific hypotheses.

Gender was almost equally split (399 females, 408 males), and ages ranged from 16 to 74 years (*M* = 35.9, *SD* = 11.3).

### Dataset 2: qualtrics panels (Brazil, Russia, the Philippines South Korea)

Dataset 2 consists of responses from 800 individuals. The survey of Dataset 1 was translated by Qualtrics into Brazilian Portuguese, Russian, Tagalog, and Hangul, omitting the death thought accessibility measure; only the 12 final items of the EDAS are included in this dataset. Qualtrics Panels then recruited participants in February 2015 to complete the survey, with a target of n = 200 completed surveys per country. To obtain these samples, a total of 2,058 participants attempted to start the survey: 37 were disqualified because they were not from the specified countries; a further 408 did not provide informed consent, and therefore did not proceed. As in Dataset 1, an attention check was included near the beginning of the survey—also the PANAS, to which participants were asked to respond by checking only “none of the above”—which 684 respondents failed; a further 129 did not complete the survey. When data collection was completed, Qualtrics then translated participants’ free-list responses into English.

Across this dataset, there were almost equal numbers of males and females (422 females, 376 males, 2 other), though the individual samples were varied: Brazil (71 females, 127 males, 2 other), South Korea (128 females, 72 males), the Philippines (113 females, 87 males), Russia (110 females, 90 males). Age ranges were similar across samples (*M* = 35.4, *SD* = 10.5): Brazil (20–73 years; *M* = 36.5, *SD* = 11.6), South Korea (19–66 years; *M* = 34, *SD* = 11.5), the Philippines (20–67 years; *M* = 35, *SD* = 9.8), Russia (20–64 years; *M* = 35.8, *SD* = 10).

### Dataset 3: lancers (Japan)

Dataset 3 consists of responses from 219 respondents. The survey of Dataset 3 is a translation of the survey of Dataset 1—by a native English speaker at the University of Oxford and a native Japanese speaker at Hokkaido University—but a few changes were introduced, as described below. The survey was uploaded to the Qualtrics platform and then advertised on Lancers (https://www.lancers.jp/)^23^, a Japanese crowdsourcing platform, in June-July 2015; participants were paid ¥324 for completing the survey. As for Database 2, we aimed for 200 completed surveys. To accomplish this, 339 began the survey; 26 failed to provide informed consent; 85 further failed to pass the attention check. Of the remaining, 9 failed to complete every section of the survey.

As in Dataset 2, Dataset 3 does not include a death thought accessibility measure. The attention check task also differed in this case: participants were asked to indicate—falsely—that they were recruited for the study via “Amazon MTurk”. Due to a miscommunication with translators, many of the scales were presented with truncated response options (e.g., from 7-points to 6). The Death Anxiety Questionnaire required responses on an 8-point scale (anchored at 1 = “Not at all” and 8 = “Very much so”) rather than a 9-pont scale from 0 to 8. Similarly, rather than a −4 (Strongly disagree) to 4 (Strongly agree) scale, the Existential Death Anxiety Scale presented the same 8-point scale as the DAQ. The questions about contact and familiarity with the dead; concern for what happens to their bodies and their locations, and for leaving instructions; were all presented with 6-point scales rather than 7-point scales. Mortuary preferences; questions about participants’ self-attributed religiosity and spirituality; questions about participation in religious activities; and questions about the quality of religious experiences were all presented with 8- rather than 9-point scales. This was a regrettable error, which was only caught after data collection was completed.

This sample comprised 93 female and 122 male participants, with 3 reporting “other” and 1 skipping the gender question. Ages ranged from 20 to 66 years (*M* = 36.6, *SD* = 8.5). Note that in this dataset, participant were asked for their age, rather than for the year in which they were born. Furthermore, ethnicity in this dataset was not an open-ended question as above, but a checklist: participant who responded by checking “Other” and specifying “Japanese” were recategorized as East Asian (n = 7).

## Data Records

All three datasets have been anonymised and are available in XLSX and CSV (non-proprietary) formats on the Open Science Framework (OSF) platform together with files of the questionnaires^14^. Abbreviation guides for variable names are also included in each XSLX file as well as in separate PDF files.

## Technical Validation

These datasets only included participants who passed attention checks and who did not skip any sections of survey (Dataset 1, 2, and 3).

As one major aim of these studies was to validate the Existential Death Anxiety scale (EDAS) and the brief form of the Supernatural Belief Scale (SBS-6), we present here preliminary reliability and convergent analyses. Further psychometric evaluation of these scales can be found elsewhere^10^.

To validate the EDAS, we inspected the correlation between EDAS and DAQ. Conte, Weiner, and Plutchik’s original validation study found that the DAQ enjoys split-half reliability of 0.76 and 0.83^20^; in Dataset 1, Guttman split-half was 0.87. Following the original validation study, we ran principal components analysis with varimax rotation to extract five factors, which explained a total of 73.5% of the variance. The factors closely but did not perfectly match those reported by Conte *et al*. (see Tables 1 and 2). Split-half reliability for the EDAS was 0.96. Horn’s parallel analysis^24,25^ (HPA) determined a single factor solution; principal axis factoring with varimax rotation found that the first factor explained 83.1% of the variance (see also Table 5 for other countries). As expected, DAQ and EDAS were highly correlated, r = 0.77, p < 0.001 (see Table 3).

**Table 1:** Summary of differences between datasets.

Dataset	Countries	Platform	N	Death Thought Accessibility	EDAS	Attention Check	Notes
Dataset 1	USA	Amazon MTurk	813	Y	Data for 17 items included	Bogus PANAS	
Dataset 2	Brazil, South Korea, Philippines, Russia	Qualtrics	800	N	Data for final 12 items included	Bogus PANAS	
Dataset 3	Japan	Lancers	219	N	Data for final 12 items included	MTurk question	Multiple measures were presented with truncated response scales.

**Table 2:** Principal axis factoring (varimax) of DAQ in Dataset 1 (n = 813).

Factor	% variance explained	Item	Factor loading
1	20.5	1. Do you worry about dying?	0.74
		2. Does it bother you that you may die before you have done everything you wanted to?	0.62
		14. Does the thought worry you that with death you may be gone forever?	0.82
		15. Are you worried about not knowing what to expect after death?	0.8
2	16.3	3. Do you worry that you may be very ill for a long time before you die?	0.82
		5. Do you worry that dying may be very painful?	0.72
		8. Does the thought bother you that you might lose control of your mind before death?	0.74
3	13.2	6. Do you worry that the persons most close to you won’t be with you when you are dying?	0.79
		7. Do you worry that you may be alone when you are dying?	0.79
4	11.9	10. Does it worry you that your instructions or will about your belongings may not be carried out after you die?	0.67
		11. Are you afraid that you may be buried before you are really dead?	0.82
		13. Do you worry that those you care about may not remember you after your death?	0.53
5	11.5	4. Does it upset you to think that others may see you suffering when you die?	0.57
		9. Do you worry that expenses connected with your dying will be a burden for other people?	0.81
		12. Does the thought of leaving loved ones behind you when you die disturb you?	0.6

**Table 3:** DAQ and EDAS split-half reliability and correlation.

	N	DAQ	EDAS	r
USA	813	0.87	0.96	0.77
Brazil	198	0.87	0.95	0.78
South Korea	199	0.86	0.94	0.74
Philippines	198	0.90	0.95	0.71
Russia	198	0.84	0.92	0.65
Japan	219	0.67	0.91	0.64

Split-half reliability for the SBS was 0.96; HPA indicated that a single factor could be extracted, and principal axis factoring with varimax rotation found that the first factor explained 85.7% of the variance (see also Table 5 for other countries). Multiple convergent validity checks were run. Participants were categorised based on their stated religious affiliations as atheist (n = 137), agnostic (n = 142), nonreligious (n = 91), or religious (n = 440); 3 participants did not answer the question. ANOVA found that stated religion predicted SBS scores, F(3, 806) = 421.47, p < 0.001. Post-hoc Tukey tests found that religious participants (M = 2.32, SD = 1.7) reported higher SBS scores than nonreligious (M = −1.09, SD = 2.5), agnostic (M = −1.02, SD = 1.4), and atheist (M = −3.31, SD = 1.3) participants; atheists reported lower scores than agnostic and nonreligious participants; and agnostic and nonreligious participants did not significantly differ (ps < 0.001). SBS scores also significantly predicted self-attributed religiosity and spirituality; perceived importance of religion to their lives; and every included category of religious behaviour (ps < 0.001; see Table 4).

**Table 4:** SBS split-half reliability and Pearson correlations.

Country	SBS	Religious	Spiritual	Importance	Pray privately	Attend services	Visit sites or shrines	Volunteer	Tell others
USA	0.96	0.77	0.81	0.67	0.79	0.58	0.42	0.46	0.54
Brazil	0.86	0.55	0.48	0.65	0.62	0.44	0.44	0.32	0.42
South Korea	0.89	0.60	0.59	0.67	0.60	0.58	0.58	0.48	0.55
Philippines	0.86	0.37	0.36	0.42	0.52	0.36	0.31	0.20	0.36
Russia	0.84	0.54	0.35	0.45	0.48	0.38	0.32	*0.09*	0.28
Japan	0.90	0.45	0.48	0.44	0.32	0.22	0.19	0.21	0.23

*n.s*.

Else, *p* < 0.001.

**Table 5:** EDAS and SBS % variance accounted for by first factor.

Country	EDAS	SBS
USA	83.1	85.7
Brazil	74.0	62.4
South Korea	72.9	71.2
Philippines	75.3	58.3
Russia	70.5	57.6
Japan	75.1	62.1

As DAQ was designed and validated in an American context, we had no *a priori* expectation that the five-factor structure would hold in any other context represented in Datasets 1 and 2. However, we did expect acceptable levels of internal reliability: with the exception of the DAQ in Japan, split-half analyses indicated high levels of internal reliability for DAQ, as well as for EDAS and SBS (see Tables 2 and 3). As in Dataset 1, DAQ and EDAS were consistently highly correlated, ps < 0.001. Correlations between SBS with religious behaviours and importance of religion were more variable, but always detectable, p < 0.001; however, SBS scores were not correlated with self-attributed religiosity and spirituality in Brazil, the Philippines, and Russia.

In summary, the two key measures—EDAS and SBS—behaved as expected, demonstrating consistently high levels of internal reliability and convergent validity with related indicators.

## ISA-Tab metadata file

Download metadata file
