# Author Correction: Death anxiety, exposure to death, mortuary preferences, and religiosity in five countries

**DOI:** 10.1038/s41597-020-0357-2

**Published:** 2020-01-08

**Authors:** Jonathan Jong, Jamin Halberstadt, Matthias Bluemke, Christopher Kavanagh, Christopher Jackson

**Affiliations:** 1 Centre for the Study of Social Cohesion, University of Oxford, Oxford, UK; 2 Department of Psychology, University of Otago, Dunedin, New Zealand; 3 GESIS—Leibniz institute for the Social Sciences, Mannheim, Germany

Correction to: *Scientific Data* 10.1038/s41597-019-0163-x, published online 21 August 2019

Following publication, it was found that due to a copying error a number of the values given in Table 4 of this Data Descriptor were incorrect. Table 4 has been corrected in both the HTML and PDF versions. The main text and underlying dataset remain unaltered.

